# The impact of 6-week flywheel eccentric training on sprint speed and change-of-direction of female basketball players

**DOI:** 10.1371/journal.pone.0335593

**Published:** 2025-10-31

**Authors:** Wuwen Peng, Wenhao Qu, Ruixiang Yan, Jiamin Xu, Hong Lin, Jian Sun, Duanying Li

**Affiliations:** 1 School of Athletic Training, Guangzhou Sport University, Guangzhou, China; 2 Guangdong Provincial Key Laboratory of Human Sports Performance Science, Guangzhou, China,; 3 Key Laboratory of Human-Computer Intelligent Interaction for Athletic Performance and Health Promotion, Guangzhou Sport University, Guangzhou, Guangdong, China; Manipal College of Health Professions, INDIA

## Abstract

Given the demands of basketball for rapid sprinting and directional changes, and the growing interest in flywheel eccentric training (FET) to enhance these abilities, this study examines a six-week FET regimen compared to barbell squat training (BST)—the control condition—on sprint and change-of-direction performance in female collegiate basketball players.Nineteen female collegiate basketball players were randomized to flywheel eccentric training (n = 9) or barbell squat training (n = 10) groups. Both groups trained twice weekly over six weeks, following a standardized warm-up protocol. The FET group performed 4× (2 + 8) maximal concentric–eccentric repetitions on a flywheel device (inertia = 0.075 kg·m²), and the BST group completed 4 × 8 back-squat repetitions at 80% of their pre-test one-repetition maximum (1RM); all sets were separated by 3-minute rest intervals. A 2 × 2 factorial analysis of variance (ANOVA) was conducted to assess pre– to post-intervention changes in sprint speed (20-m sprint) and change-of-direction ability (505 agility test and lane agility test).The results of the analysis of variance indicated that in the FET group, the main effect of time and the interaction effect were significant for the 20m sprint and 505 agility tests (p < 0.05), while the group main effect was not significant (p > 0.05). However, no significant effects were observed for the lane agility test (p > 0.05). In the BST group, there were no significant effects regarding the main effect of time, the group main effect, or the interaction effect in any of the tests (p > 0.05). In collegiate female basketball players, six weeks of moderate-inertia (0.075 kg·m²) flywheel eccentric training elicited superior improvements in linear sprint speed and rapid direction changes ability compared to an equivalent barbell squat regimen.

## Introduction

The high-intensity and fast-paced nature of competitive basketball requires athletes to possess exceptional short-distance acceleration and change-of-direction abilities to respond to rapid offensive and defensive transitions [1,2]. Research indicates that the average duration of a game is approximately 4520 ± 130 seconds, with specific game actions accounting for about 41%, including an average of 44 ± 7 jumps, 94 ± 16 direction changes, and 55 ± 11 sprints on the court [1]. These movements mainly occur under dynamic game situations [3], making them critical determinants of match outcomes [4]. In comparable sprint and change-of-direction (COD) tasks, women more frequently exhibit reduced knee flexion and increased knee valgus [5],which constitutes a “high-risk” kinematic pattern consistent with the higher burden of ankle sprains and knee injuries (including ACL) in collegiate women’s basketball [6],thereby underscoring the need to prioritize lower-limb eccentric braking, neuromuscular control, and stretch–shortening cycle (SSC) efficiency in training [7].

Previous research shows that eccentric strength training enhances short-distance sprinting and rapid change-of-direction performance by optimizing neuromuscular control [8], boosting muscle strength [9], and enhancing the SSC efficiency [10].Eccentric training can be categorized by load source and execution mode, with common forms including Accentuated Eccentric Loading (AEL), Accelerated Eccentric Loading (ACEL), and Inertial Resistance Training (IRT) (Handford et al., 2022). Unlike AEL, which uses external weights, and ACEL, which increases eccentric load by enhancing movement speed, Flywheel Eccentric Training (FET)—a form of IRT—generates resistance through the inertia of a rotating flywheel [11]. In FET, the athlete actively accelerates the flywheel during the concentric phase, while the eccentric phase delivers a continuous, dynamic, and self-regulated supramaximal stimulus via the flywheel’s returning inertia [12].

Building on this mechanical distinction,FET is characterized by its adaptive resistance and targeted eccentric overload [13], whereas traditional barbell squat training (BST) elicits greater concentric but lower eccentric muscle activation [14]. Conversely, FET achieves eccentric peak power with lower metabolic cost and muscle activation [14],and the increased eccentric load subsequently enhances concentric activation [13]. However, evidence for the effects of flywheel eccentric training (FET) on sprint and COD performance is inconsistent: in female basketball players, O’Brien et al. [15] reported improvements in 10-m sprint performance but no significant changes in COD, whereas in professional women’s soccer, six weeks of flywheel squats significantly increased maximal strength without conferring additional gains in sprint or COD [16]. Meanwhile, systematic reviews also note a general paucity of studies involving female samples and substantial heterogeneity in intervention parameters and outcome measures [17].These discrepancies may reflect differences in the intervention timing, protocol design, and whether FET served as a primary or supplementary training modality, complicating consistent evaluation of itsimpact on sprinting and COD abilities.

Given the current lack of clarity regarding the effects of FET on sprint speed and COD performance in basketball players—particularly in female basketball players—further investigation is warranted [17].This study seeks to compare the effects of FET and BST on sprint speed and change-of-direction capabilities among female athletes. Participants in the BST group performed conventional back squats at 80% of their pre-assessed one-repetition maximum (1RM). The FET group trained using a flywheel device with a moment of inertia of 0.075 kg·m², which provided continuous and adaptive eccentric overload through a combination of concentric acceleration and resisted eccentric braking.The hypothesis posits that FET will significantly improve lower limb sprint speed and COD in collegiate female basketball players, thereby augmenting their rapid response and tempo transition capabilities during competitive play.

## Materials and methods

### Participants

This study employed a randomized parallel-controlled trial design. The required sample size was calculated using G*Power 3.1 software, yielding a minimum required sample size of 18 participants, with effect size (ES), alpha (α), and power set at 0.5, 0.05, and 0.8 [18]. Considering a potential dropout rate of 10%, a total of 24 female college basketball players were subsequently recruited. All participants were members of the basketball team at Guangzhou Sport University, certified national second-level athletes, and had previously competed in the China University Basketball Association League (CUBAL). Each participant had at least five years of systematic basketball training experience. The recruitment period for participants in this study was from April 8, 2024 to April 15, 2024.Inclusion criteria for participants were as follows: (1) female college basketball players with at least 3 years of basketball training; (2) no sports injuries in the past six months; (3) at least 2 years of resistance training experience.

**Fig 1:**
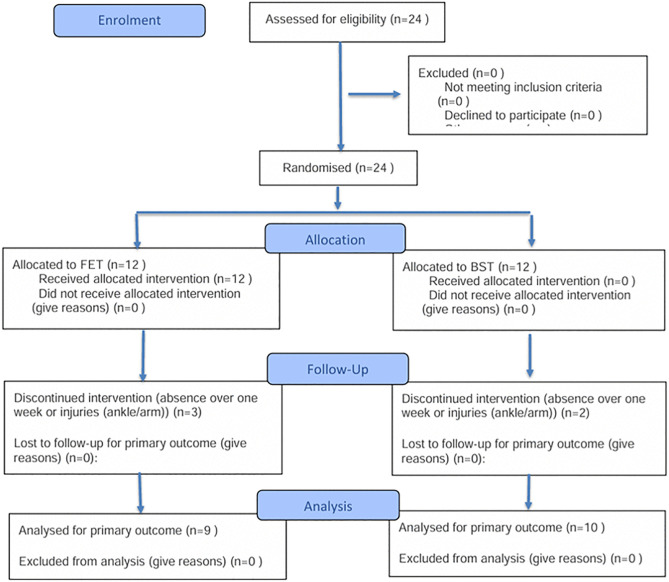
CONSORT participant flow diagram.

After completing familiarization and pre-testing, participants were randomly allocated to either the FET group or the BST group (Fig 1). Randomization was performed using opaque, sealed envelopes prepared by an independent researcher. Group assignments were carried out by designated personnel following baseline assessments. Although participants were necessarily aware of their assigned training condition due to the nature of the interventions, they were not informed of the study hypotheses or the expected differences between groups, Participants were not informed of the specific study hypotheses or the expected differences between the two training interventions. All participants were instructed to complete the training tasks with maximal effort, regardless of group assignment. Allocation was concealed during randomization stage, and all outcome assessments and data analyses were conducted by blinded investigators to minimize potential bias. We followed the CONSORT guidelines for randomized trials; see S1 File.

Five participants withdrew from the training process (2 due to absence for more than one week; 3 due to ankle or arm injuries sustained in competitions outside the intervention). Ultimately, 19 participants were retained for statistical analysis (age: 20.47 ± 2.27 years; height: 167.26 ± 4.16 cm; weight: 61.56 ± 7.07 kg), comprising the experimental group (FET, n = 9) and the control group (BST, n = 10). Basic information is presented in Table 1.

**Table 1:** Basic information of the participates.

Variable	FET (n = 9)	BST (n = 10)
**Age (year)**	20.7 ± 2.0	20.2 ± 2.6
**Height (cm)**	167.9 ± 4.7	166.5 ± 3.5
**Body mass (kg)**	61.9 ± 4.7	60.1 ± 7.6

Note: FET = flywheel eccentric training; BST = Barbell squat training.

All participants were adults (aged 18 years or older) and received a detailed explanation of the study procedures, potential risks, and anticipated benefits prior to enrollment. Each participant signed a written informed consent form before participating in the study.All procedures involving human subjects compliedwith the Declaration of Helsinki, and ethical approval was obtained from the Institutional Review Board of Guangzhou Sports University (Approval No. 2023LCLL-45). This study was approved by the Chinese Clinical Trial Registry on 08/04/2024 (Registration number ChiCTR2400082807).

### Procedures

**Fig 2:**
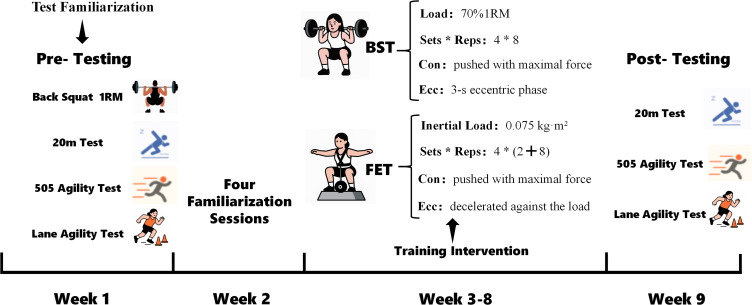
Experimental intervention flowchart.

Participants underwent a 9-week experimental protocol comprising 2 weeks of familiarization and pre-testing, 6 weeks of intervention, and 1 week of post-testing (Fig 2). The pre-test occurred 48 hours before the intervention, and the post-test took place 48 hours after the final training session. All performance tests were conducted over two consecutive afternoons to minimize circadian influences on performance. On the first afternoon, participants completed the 20 m sprint test and the 505 agility test sequentially, with 3-minute passive recovery periods between trials and tests to ensure adequate recovery. The Lane agility test was performed on the second afternoon at the same time of day. These tests are widely recognized as valid and reliable for assessing sprint speed and COD performance in basketball players [19–21].

To ensure participants were adequately familiarized with the course content and could safely participate in the experiment, the following measures were undertaken: (1) Detailed records of participants’ height, weight, and waist circumference were collected through anthropometric measurements to ascertain the requisite barbell height and belt size for training. (2) Participants were educated on the standardized operating procedures and technical essentials for FET and BST to guarantee consistency and standardization in the experimental process. (3) A maximum strength-related test was conducted during the final familiarization session, and the results were used to estimate the training load for 80% of the one-repetition maximum (1RM) for the barbell squat, thereby facilitating the formulation of a suitable training plan.

Considering that novice trainers without prior experience in FET necessitate a period of adaptation, research indicates that at least two training sessions are needed to achieve stable performance levels [22]. Therefore, four familiarization sessions were scheduled to ensure participants acclimated to the training equipment and mastered the correct squat technique. All tests and training sessions were conducted in the Digital Physical Training Laboratory of Guangzhou Sport University, under the full supervision of three CSCS-certified staff members to ensure technical accuracy and safety. Throughout the experimental period, participants were instructed to refrain from engaging in other high-intensity training and to avoid the consumption of alcohol, caffeine, and other ergogenic aids that could potentially enhance neural excitability. The final study protocol is provided in S2 File.

### Training routine

Research indicates that isometric peak torque and muscle soreness typically return to levels close to baseline within 48–72 hours after flywheel training [23]. Consequently, during the intervention period, all athletes engaged in five basketball-specific training sessions per week, each lasting approximately 120 minutes. To help participants adapt to the load and maintain a stable weekly volume of technical and tactical training, the schedule comprised three technical-tactical sessions on Tuesday, Friday, and Saturday, and two physical conditioning sessions on Monday and Thursday.

Prior to the formal experimental intervention, all participants underwent a standardized warm-up following the RAMP principles proposed by the National Strength and Conditioning Association (NSCA) [24]. The warm-up included the following components: 2 minutes of foam rolling, 3 minutes of dynamic stretching (including calf raises, cradle stretches, the world’s greatest stretch, and A-skips), 3 minutes of neuromuscular activation (incorporating T-splits, crossover steps, and continuous vertical jumps followed by sprints), and 2 sets of 10 bodyweight half squats.

BST: Participants in the BST group utilized a free squat rack for barbell squat training. The intensity was determined based on a percentage of the one-repetition maximum (1RM) derived from previous testing. The NSCA recommends training at 80% of 1RM to optimize lower body maximal strength. Therefore, the BST group trained at an intensity of 80% of their 1RM, performing a total of 4 sets of 8 repetitions, interspersed with 3 minutes of rest between sets.

FET: The training load for the FET group was informed by the findings of Sabido, Mikic, and Corratela et al. [25–27], which indicate that an inertia of 0.075 kg·m² generates substantial eccentric loads and greater contraction velocities, positively influencing strength levels. Thus, the FET group trained at an inertia of 0.075 kg·m², performing 4 sets of 8 repetitions with 3 minutes of rest between sets. Prior to each training set, participants were required to perform 2 repetitions of FET to accelerate the flywheel; during the eccentric squat phase, maximal control was maintained until the knee flexed to 90 degrees. Additionally, the experiment utilized Desmotec equipment (Italy), with average concentric power monitored in real-time via Bluetooth connection to a compatible client application (available for both iOS and Android), utilizing the proprietary kMeter software system. Specific training details are presented in Table 2.

**Table 2:** Training program and training volume.

Training Structure	Group	Training Method	Sets * Reps/ Rest Interval	Load
**Warm-up**	FET and BST Groups	Foam Rolling Dynamic Stretching Neural Activation 2 sets of 10 Half Squats	10 minutes	/
**Training Intervention**	FET Group	Flywheel Eccentric Training	4* (2 + 8) /3minutes	0.075 kg·m²
	BST Group	Barbell Squat Training	4*8/3minutes	80%1RM
**Stretching**	FET and BST Groups	Foam Rolling for Relaxation	10-15minutes	/

### Outcome measures

Speed Test: A 20 m sprint test was conducted utilizing a Brower Timing Systems wireless timer on an indoor rubber track. Participants were instructed to demonstrate maximal effort during the sprint. Each participant completed three times, with a 3-minute rest interval between each trial. The shortest recorded time was recorded as the final result, accurate to 0.01 seconds. The test-retest reliability for the 20 m sprint was calculated as ICC = 0.954, 95% CI: 0.886–0.982, and CV = 4.6%.

COD Test: Two agility tests were conducted, including the lane agility test and the 505 agility test. For the lane agility test, cones were placed at each corner of the lane. Participants sprinted from the lower left corner of the free-throw line to the baseline, shuttled laterally to the upper-right corner, backpedalled to the lower-right corner, shuttled laterallyto the lower-left corner, and returned along the same route to complete one circuit. Each participant completed two circuits, and times were recorded via a wireless timing system. The test-retest reliability for the lane agility test was calculated as ICC = 0.79, 95% CI: 0.56–0.915, with a CV of 3.8%.

The 505 agility test primarily assesses an athlete’s ability to accelerate, decelerate, and change direction, according to the methodology established by Stojanović et al [28]. The test-retest reliability for the 505 agility test was calculated as ICC = 0.604, 95% CI: 0.225–0.825, with a CV of 5%. Each test was conducted three times, with a 3-minute rest interval between trials to ensure adequate recovery. The shortest recorded time was used, with an accuracy 0.01 seconds.

### Statistical analyses

Descriptive statistics are presented as mean ± standard deviation (SD), with significance levels represented by p-values, where p < 0.05 indicates a statistically significant difference. Statistical analyses were performed using SPSS version 23.0. The Shapiro–Wilk test and Levene’s test were applied to assess the normality and homogeneity of variance, respectively, for the three primary performance outcomes prior to conducting inferential analyses. In the per-protocol (PP) analysis, a two-way repeated measures ANOVA was conducted to assess the interaction effect between group (FET vs. BST) and time (pre- and post-test), which served as the primary analysis to determine intervention effects. Post-hoc analysis was performed utilizing the Sidak method for each outcome measure. For the three outcome measures, we applied a Bonferroni correction (α′ = 0.05/3 ≈ 0.017) to control the family-wise type I error rate; all paired comparisons or group × time interaction tests were considered significant at p < 0.017 [29]. As a sensitivity analysis, we additionally implemented an intention-to-treat (ITT) linear mixed-effects model (LMM) that included all randomized participants assigned to FET or BST, irrespective of protocol adherence or loss to follow-up. The test-retest reliability of the 20 m sprint, 505 agility test, and Lane agility test was evaluated using coefficients of variation (CV) [30] and intraclass correlation coefficients (ICC) [31] with 95% confidence intervals (CIs). An ICC value below 0.5 indicates poor reliability, while values between 0.5 to 0.75 indicate moderate reliability, values from 0.75 to 0.9 indicate good reliability, and values above 0.90 indicate excellent reliability. An CV less than 10% is regarded as reliable.Effect sizes for group differences were assessed using partial eta squared ($\eta_{p}^{\text{2}}$), which represents the proportion of variance explained by a specific factor or interaction relative to the variance not accounted for by other effects in the model.Effect sizes were interpreted according to the following thresholds:small (0.01 ≤ $\eta_{p}^{\text{2}}$ ≤ 0.06), medium (0.06 ≤ $\eta_{p}^{\text{2}}$ < 0.14), and large ($\eta_{p}^{\text{2}}$ ≥ 0.14) [32]. Cohen’s d was employed to calculate the effect sizes of the intervention, with classification standards defined as follows: effect sizes less than 0.2 are considered trivial, 0.2 to 0.5 indicate a small effect, 0.5 to 0.8 indicate a moderate effect, and ≥0.8 indicate a large effect [33].

## Results

All three outcome variables (20-m sprint, 505 agility, and Lane test) showed approximately normal distributions at both pre- and post-training (Shapiro–Wilk W = 0.878–0.936, all p > 0.05)(S1 Fig). Levene’s test revealed significant variance heterogeneity for pre-20 m (F = 5.359, p = 0.033), pre-Lane (F = 7.903, p = 0.011) and post-505 (F = 1.794, p = 0.012), while variances were equal for post-20 m (F = 2.199, p = 0.156), pre-505 (F = 0.471, p = 0.502) and post-Lane (F = 8.040, p = 0.198) (S2 Fig). Therefore, we applied standard repeated-measures ANOVA for the comparisons with equal variances, and used Welch’s correction for those comparisons exhibiting variance heterogeneity. In addition, 5/24 participants (20.8%) did not complete the post-test; attrition was comparably distributed between groups (FET: 3/12 = 25.0%; BST: 2/12 = 16.7%). Baseline values of the primary outcomes were comparable between completers and dropouts (20 m: Welch t = 1.79, p = 0.091; 505: t = 0.31, p = 0.765; lane agility: t = 0.15, p = 0.880), suggesting a weak association between missingness and baseline performance. To mitigate potential inference bias due to attrition, we conducted an intention-to-treat sensitivity analysis using a linear mixed-effects model with REML under the Missing-At-Random (MAR) assumption; the findings were directionally consistent with the per-protocol analyses. Taken together, these results indicate that the main conclusions are reasonably robust to plausible assumptions about the missing-data mechanism (Raw data are available in S1 Table).

### Sprint speed

The results of the repeated measures ANOVA showed a significant main effect of time for the 20-m sprint between the FET group and the BST group (p < 0.01, $\eta_{p}^{\text{2}}$ = 0.88). However, the main effect of group was notsignificant (p > 0.05, $\eta_{p}^{\text{2}}$ = 0.04), whereas the time × group interaction was significant (p < 0.01, $\eta_{p}^{\text{2}}$ = 0.40). When comparing pre- to post-training performance, the FET group improved their 20-m sprint time by 2.46% (ES = 0.90, large), whereas the BST group improved by 1.32% (ES = 0.49, medium). In the intention-to-treat linear mixed-effects model, the 20-m test results were directionally and statistically consistent with the PP analysis (ES = 0.46, p < 0.01).

### COD

**Fig 3:**
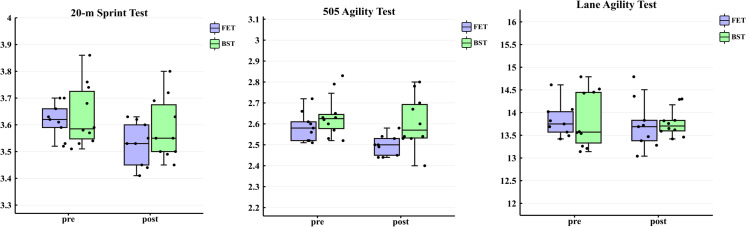
Comparison of pre- and post-test performance between FET and BST groups.

For the 505 agility test, a significant main effect of time was observed between the FET and BST groups (p < 0.01, $\eta_{p}^{\text{2}}$ = 0.50), while the main effect of group was not significant (p > 0.05, $\eta_{p}^{\text{2}}$ = 0.19), and the time × group interaction was significant (p > 0.017, $\eta_{p}^{\text{2}}$= 0.22). When comparing pre- to post-training performance, FET elicited a 3.46% reduction in time (ES = 0.97, large), compared to a 1.06% reduction for BST (ES = 0.30, small). In the intention-to-treat linear mixed-effects model, the 505 agility test results were directionally and statistically consistent with the PP analysis (ES = 0.80, p < 0.05). In contrast, the main effects of group, time, and interaction for the agility test in the lane demonstrated no significant differences (p > 0.05 for all), When comparing pre- to post-training performance, FET changed by 0.62% (ES = 0.20, small) and BST by 0.40% (ES = 0.15, trivial)(Fig 3, Table 3). In the intention-to-treat linear mixed-effects model, the Lane agility test results were directionally and statistically consistent with the PP analysis (ES = 0.06,p > 0.05).

**Table 3:** Sprint speed and change-of-direction ability.

Project	Group	PRE	POST	Change%	Cohen’s d	Group			Time			Group × Time		
						F	P	$\eta_{p}^{2}$	F	P	$\eta_{p}^{2}$	F	P	$\eta_{p}^{2}$
**20m sprint test**	FET	3.62 ± 0.07	3.53 ± 0.08*	2.46%	0.90	0.74	0.40	0.04	125.24	0.00**	0.88	11.17	0.00**	0.40
	BST	3.64 ± 0.12	3.59 ± 0.12*	1.32%	0.49									
**505 agility test**	FET	2.59 ± 0.07	2.50 ± 0.05*	3.46%	0.97	4.09	0.06	0.19	16.85	0.00**	0.50	4.65	0.04*	0.22
	BST	2.64 ± 0.10	2.61 ± 0.13	1.06%	0.30									
**Lane agility test**	FET	13.82 ± 0.37	13.73 ± 0.64	0.62%	0.20	0.04	0.85	0.00	0.40	0.56	0.02	0.00	0.93	0.00
	BST	13.85 ± 0.63	13.77 ± 0.31	0.40%	0.15									

Note: * indicates a significant difference within the group, p < 0.05; ** indicates a highly significant difference within the group, p < 0.01; FET = flywheel eccentric training; BST = barbell squat training.

## Discussion

The aim of this study was to compare the effects of a 6-week FET program with a BST program regarding 20-m sprint performance, 505 agility test results, and Lane agility test outcomes in female collegiate basketball players. The findings suggest that the FET group demonstrated significantly greater improvements in both the 20-m sprint and the 505 agility test compared to the BST group, with a particularly large effect size observed in the 20-msprint (ES = 0.90). However, neither group exhibited significant improvements in the Lane agility test.

This study shows that the implementation of moderate-inertia FET (0.075 kg·m²)significantly improved the sprinting speed of female collegiate basketball players, surpassing the performance of the BST group. This finding aligns with the research conducted by Izquierdo et al [34]. Adequate lower-limb strength enhances initial propulsion and optimizes ground-reaction force utilization, improving sprint efficiency and speed [35]. FET has been shown to effectively enhance muscle mass and strength in the lower limbs of female athletes [36], It selectively recruits high-threshold motor units during eccentric loading, increasing overall force capacity [37]. Furthermore, greater eccentric loading boosts elastic energy storage via stretch-reflex mechanisms [38], This elevates neuromuscular activation in subsequent concentric actions, optimizes fast-twitch fiber recruitment,and ultimately improves sprint speed [39].

However, other studies report conflicting effects of FET on sprint performance.. For instance, Arede et al. [40] applied a 6-week FET with 0.0315 kg·m² inertia in adolescent female athletes and found no significant changes in 5-m or 10-m sprint times..Possible reasons include the athletes’ developmental stage and the lower inertia (0.0315 kg·m²). Another contributing factor could be the difference in test distances; research suggests that longer sprint distances activate the posterior leg muscles more effectively, which may hinder the observation of significant training effects at shorter distances [41].

Regarding COD, the FET group exhibited significantly greater improvements in the 505 agility test compared to the BST group. This aligns with Asencio et al. [42], who reported a 7–10% improvement in the 505 agility test after flywheel training protocols involving 29 athletes, thereby highlighting the effectiveness of FET in enhancing COD performance. FET’s adaptable eccentric overload likely confers greater specificity for COD performance [43]. COD depends on multiple factors, especially maximum lower-limb eccentric force [44],because during the braking phase, the quadriceps must generate sufficient eccentric torque to decelerate and stabilize the body, thus setting up a rapid re-acceleration into a new direction [45]. Mechanistically, FET exploits the rotational inertia of a flywheel to impose continuous, supramaximal eccentric resistance throughout the entire range of motion.This stimulus induces microdamage to muscle fibers, activating satellite cells, and stimulating the mTOR signaling pathway to enhance protein synthesis and promote cross-sectional hypertrophy [46].Simultaneously FET recruiting high-threshold motor units and driving peripheral neural adaptations such as improved motor-unit synchronization and firing rate to elevate muscle activation in both eccentric and ensuing concentric phases [36], and, in addition, by increasing muscle–tendon stiffness and augmenting elastic-energy storage and release, it potentiates the stretch–shortening cycle and post-activation potentiation to further boost dynamic athletic output [41].

However, Pecci et al. [16] found that a 6-week FET with an inertia of 0.025 kg·m² did not enhance the sprinting and change-of-direction abilities of female soccer players, contrasting with the findings of the current study. This discrepancy may be attributed to the higher inertia utilized in the current study (0.075 kg·m²). Additionally, Jones et al. [47] found a strong correlation between higher eccentric force and COD ability in elite female soccer players.This suggests that greater eccentric force helps athletes handle larger loads at high velocities, which may explain our observed COD improvements.

Interestingly, neither group exhibited significant improvements in the Lane agility test. The Lane agility test emphasizes footwork flexibility and overall control. Test performance is influenced by training factors but is also closely linked to the athlete’s physical condition and sport-specific skills. A singular training modality or method is unlikely to produce substantial improvements in Lane agility. This suggests that moving from general to sport-specific agility requires varied stimuli and longer training duration for noticeable gains.

The present study has several limitations that should be acknowledged.This study has several limitations. First, the sample included only collegiate female basketball players without stratification by playing position, preventing analysis of how guards, forwards, and centers may differentially respond to flywheel eccentric training. Second, the modest sample size may have limited statistical power for some outcomes, possibly explaining non-significant findings; future research should use larger cohorts or alternative designs to confirm these results. Finally, the lack of a no-training control group complicates attribution of observed changes to the intervention rather than to familiarization or other external factors.

## Conclusion

This study demonstrates that a six-week FET intervention using moderate inertia (0.075 kg·m²) confers superior adaptations in 20 m sprint velocity and 505 agility performance compared to an equivalent BST regimen in collegiate female basketball athletes, whereas neither protocol meaningfully altered lane-agility outcomes. These data indicate that moderate-inertia FET may serve as a more potent stimulus for enhancing linear speed and rapid direction changes in this population.However,the generalizability of our findings is constrained by a homogeneous sample lacking positional stratification, the modest sample size, and absence of a true no-training control. Future investigations should stratify participants by playing position, compare multiple inertia loads, and include rigorous control conditions to validate and elucidate the mechanistic underpinnings of FET’s effects on basketball-specific performance domains.

## Supporting information

S1 Fig Q-Q plots of three outcome measures at baseline and post-intervention. (TIF)

S2 Fig Boxplots of three outcome measures at baseline and post-intervention. (TIF)

S1 File CONSORT 2025 checklist item description. (PDF)

S2 File Human experimental ethics inspection of GuangZhou Sport University. (PDF)

S3 File Human experimental ethics inspection of GuangZhou Sport University (Original version). (PDF)

S4 File Experimental protocol. (PDF)

S5 File Experimental protocol (Original version). (PDF)
