# Supplementary material for: The impact of 6-week flywheel eccentric training on sprint speed and change-of-direction of female basketball players
Source: PLoS One. 2025 Oct 31;20(10):e0335593. doi: 10.1371/journal.pone.0335593 (PMC12578200; doi:10.1371/journal.pone.0335593)
Supplement: S3 File — (PDF) [file pone.0335593.s005.pdf]

广州体育学院人体实验伦理审查表

Human Experimental Ethics Inspection of GuangZhou Sport University

审批编号（ID Number）: 2023LCLL-45

项目名称<br>Project name	飞轮离心深蹲训练对女子篮球运动员专项弹跳能力影响的研究	项目来源<br>Source of project	无
项目负责人<br>Project director	李端英	实验方案<br>Experimental scheme	（附件说明）
研究对象<br>Objects	（年龄、性别、健康状况、人数、职业等。）<br>年龄：18-23 岁<br>性别：女<br>健康状况：优良<br>人数：19<br>职业：学生		
实验要点<br>Outline of experiments	（包括实验周期、干预方法、观测手段和指标、涉及人体的取样或部位等。）<br>实验周期：6 周<br>实验频率：每周 2 次<br>干预方法：飞轮离心深蹲训练与杠铃深蹲训练<br>观测手段：干预前后进行指标测试<br>测试指标：20m 跑、5-0-5 动作敏捷测试、3/4 跑、禁区敏捷性测试、CMJ、SJ、DJ、助跑双脚起跳摸高、大腿围度、深蹲 1RM、卧推 1RM		
审查内容<br>Contents of inspection	1．研究方案<br>2．知情同意书<br>3．需由研究对象填写的表格和问卷<br>4．研究人员履历及联系方式		

申请者声明<br>Announcement of applicant	我将自觉遵守人体实验的伦理原则，随时接受伦理委员会的监督与检查，如违反规定，自愿接受处罚。<br>I will abide by the rules of human experimental ethics,accept the supervision and inspection of ethics committee, and accept the punishment if any infringement.<br><br>声明人签名(Signature): [illegible]<br>2023 年 2 月 7 日
审查结果<br>Results of inspection	广州体育学院伦理委员会审查意见：<br><br>☑同意 （Agree）<br>○修改后同意（Agree after revised）<br>○修改后重审（Retrial after revised）<br>○不同意 （Disagree）<br>○暂停或终止实验（Suspension or termination）<br><br>主任委员（签名）：[illegible]<br>伦理委员会（盖章）：广州体育学院伦理审查委员会<br>2023 年 9 月 11 日

说明：

1. 审批编号须审查通过后，由伦理委员会填写；
2. 申请人若为在校研究生，导师为项目负责人；
3. 审查材料一式两份，相关审查资料随本表一并递交。
