# Supplementary material for: The impact of 6-week flywheel eccentric training on sprint speed and change-of-direction of female basketball players
Source: PLoS One. 2025 Oct 31;20(10):e0335593. doi: 10.1371/journal.pone.0335593 (PMC12578200; doi:10.1371/journal.pone.0335593)
Supplement: S4 File — (PDF) [file pone.0335593.s006.pdf]

Research Proposal on the Impact of 6-Week Flywheel Eccentric Training on Sprint Speed and Change of Direction in Female Basketball Players

1. Statement of Integrity

This study guarantees that operations are strictly conducted in accordance with experimental protocols and that data recordings are authentic. There are no conflicts of interest.

2. Research Title

The Impact of 6-Week Flywheel Eccentric Training on Sprint Speed and Change of Direction in Female Basketball Players

3. Funding Source

Self-funding

4. Research Timeline

（1）April 15, 2024 – June 15, 2024: Testing and intervention phase, data collection

（2）June 16, 2024 – December 30, 2024: Data collection, statistical analysis, drafting and submission of the paper for publication

5. Research Background

The high-intensity, fast-paced nature of competitive basketball requires athletes to possess exceptional short-distance acceleration and change-of-direction abilities to adapt to the rapid offensive and defensive rhythms of the game. Research indicates that the average duration of a game is approximately 4520 ± 130 seconds, during which "specific actions" account for about 41%, including 44 ± 7 jumps, 94 ± 16 direction changes, and 55 ± 11 sprints. These actions predominantly occur in dynamic game situations and are critical not only during rapid transitions but also as decisive factors in key moments of the match. Therefore, sprinting and change-of-direction capabilities are particularly important.

Athletes' exceptional sprinting and change-of-direction abilities depend on flexible neuromuscular coordination, superior muscular strength, and good joint control. Existing studies have shown that eccentric strength training can effectively enhance short-distance sprinting and rapid change-of-direction capabilities by optimizing neuromuscular control, increasing muscular strength, and improving the stretch-shortening cycle (SSC) efficiency. Among various eccentric training methods, Flywheel Eccentric Training (FET) is widely recognized for providing greater loads

during the eccentric phase of resistance training.

FET is a training method that generates resistance through a rotating flywheel, characterized by adaptive resistance and eccentric overload. It has been found to outperform Barbell Squat Training (BST) in enhancing performance-related capabilities, such as vertical jump and sprint speed. While BST exhibits higher activation during the concentric phase, FET achieves greater peak force during the eccentric phase with lower metabolic costs and muscle activation. Furthermore, the increased load during the eccentric phase indirectly maximizes activation during the concentric phase. However, existing research on the effects of FET on sprinting and change-of-direction abilities remains controversial. For instance, studies by Pecci and Izquierdo reported opposing effects, which may be attributed to factors such as gender differences, athletic experience, and variations in inertial loads. These factors complicate the unified evaluation of FET's impact on sprinting and change-of-direction abilities.

Given the unclear effects of FET on sprint speed and change of direction in female athletes, this study aims to compare the effects of FET and BST on these abilities in female basketball players. The hypothesis is that FET will significantly improve lower limb sprint speed and change-of-direction capabilities in college female basketball players, thereby enhancing their quick response and transition abilities during competitions.

6. Research Objectives

As a novel strength training method, flywheel eccentric squat training can provide adaptive resistance and eccentric overload. This study aims to investigate how the effects of flywheel eccentric training on basketball performance differ from those of the commonly used barbell squat training among basketball athletes. Specifically, it will explore whether using a flywheel training device for squat exercises is more effective than traditional barbell squat training in enhancing basketball performance.

7. Significance and Value of the Research

As contemporary competitive sports approach the limits of human performance, the discovery of superior training methods can help athletes maintain high levels of

competitive capability and potentially break through existing barriers. This study is grounded in training practice and builds on previous research, utilizing advanced instruments and scientific testing methods to investigate the impact of flywheel eccentric training versus barbell squat training on the performance of female basketball players. Through experimental data, it aims to demonstrate the differences between the two training methods, providing valuable insights for coaches and athletes in their training practices and thereby effectively enhancing basketball performance and elevating athletic levels.

8. Research Hypotheses and Variables

Research Hypotheses:

Flywheel training can significantly improve the 20m sprint performance of female basketball players.

Flywheel training can enhance the performance of female basketball players in the 505 agility test and the restricted area agility test.

9. Inclusion and Exclusion Criteria

Inclusion Criteria:

All participants are over 18 years of age.

At least three years of experience in basketball.

Participants are from the basketball team of the Sports Training College.

Participants voluntarily join the study, sign informed consent, and demonstrate good compliance with follow-up interventions.

Exclusion Criteria:

Low compliance.

Injury leading to loss of follow-up or withdrawal during the study.

Failure to cooperate.

10. Study Design

A parallel, prospective, two-arm randomized controlled trial.

11. Sample Size Estimation

A total of 20 participants will be recruited, with 19 included in the final statistical analysis: 9 in the experimental group and 10 in the control group.

12. Randomization and Concealment Methods

Random grouping will occur after baseline testing, using SPSS to randomly assign participants to either the FET or BST group. Neither participants nor researchers will know which group they are assigned to. A random number table will be used for simple randomization, and the random allocation sequence will be generated by a second researcher, with eligibility determined by a third researcher. After grouping, participants will be informed of their group tasks by their specialized coach and advised not to engage in additional resistance training during the experiment, while normal basketball training is permitted.

13. Measurement Indicators

20m sprint, 505 agility test, restricted area agility test.

14. Definition of Participant Validity

Validity Definition: Participants who have signed informed consent, passed screening, demonstrated good compliance, and completed all intervention training as per the protocol.

Withdrawal Definition: Participants who signed informed consent but request to withdraw before, during, or after the intervention follow-up.

Exclusion Definition: Participants who signed informed consent but are found to not meet inclusion criteria during monitoring or after the study, or those who did not comply with training requirements.

Loss to Follow-Up Definition: Participants who cannot be contacted despite multiple attempts before the project ends.

Contamination Definition: All participants will be involved in additional basketball training, which has been accounted for.

Termination Definition: Cases where the project leader or researchers decide to discontinue intervention based on various factors, or where adverse events are discovered.

Suspension Definition: Participants who express doubts or intentions to withdraw after signing informed consent.

15. Definitions and Management of Adverse Events and Reactions

Adverse Event Definition: Any unfavorable event occurring between informed consent and the end of follow-up, regardless of causality.

Adverse Reaction Definition: Any harmful, non-expected response occurring during training or observation that is causally related to the intervention.

Identification Methods: Harmful, unexpected events causally related to the intervention are classified as adverse reactions; others are classified as adverse events.

Management Protocol: All adverse events or reactions will be accurately recorded, including details of occurrence, severity, duration, and measures taken.

16. Recruitment of Participants

Recruitment Location: Digital Sports Laboratory, Guangzhou Sport University.

Recruitment Methods: Creating recruitment posters, using WeChat, and coordinating with team coaches.

Screening Process:

Athletes meeting inclusion criteria are selected for preliminary screening.

Functional movement screening with no positive cases, scoring ≥14.

Strict screening according to inclusion/exclusion criteria.

Final selection based on willingness to participate and signing of informed consent.

Screening Personnel: Conducted by independent third-party personnel.

17. Collection of General Information from Participants

Data Collection Personnel: Graduate students, including Xu Jiamin.

General Information Content:

Age, height, weight, BMI, injury history, training history, athlete level.

Body composition metrics.

Baseline indicators and performance variables.

18. Statistical Analysis Methods

Statistical analyses will be performed using SPSS 23.0. Independent samples t-tests will compare baseline differences between groups. The Shapiro-Wilk test will assess normality, with Bonferroni post hoc tests for repeated measures ANOVA. Mauchly's test will check for sphericity. If normality is not met, the Scheirer–Ray–Hare test will be used. If significant interactions exist, simple effects analysis will follow; if

not, main effects will be analyzed using paired t-tests for within-group differences and independent t-tests for between-group differences. A one-way random effects model will evaluate test-retest reliability using CV and ICC. An ICC value <0.5 indicates poor reliability, 0.5–0.75 indicates moderate reliability, 0.75–0.9 indicates good reliability, and >0.90 indicates excellent reliability. Partial eta squared (η_p^2) will assess effect sizes for group differences, categorized as small ($0.01 \leq \eta_p^2 \leq 0.06$), medium ($0.06 \leq \eta_p^2 < 0.14$), or large ($\eta_p^2 \geq 0.14$).

29. Participant Management Protocol

All participants must sign a written informed consent form before intervention.

Participants will be screened based on inclusion and exclusion criteria.

Blinding and protection of participant privacy and safety will be ensured.

20. Specimen Management Protocol

This study does not involve sample collection from participants.

21. Drug and Equipment Management Protocol

This study does not involve drug interventions.

22. Data Management Protocol

All data will be backed up in real-time, with separate entries into spreadsheets managed by a third party. The database will be managed by Xu Jiamin.

23. Data Security and Composition of the Monitoring Committee

Data will be managed by a third party not involved in subsequent research, with oversight from the ethics committee.

24. Research Team

The team consists of the principal investigator, participating researchers, manuscript writers, evaluators, data collection managers, statistical analysts, and data verification personnel.

25. Intellectual Property

All intellectual property arising from this research belongs to Guangzhou Sport University, with authorship based on contributions.

26. Publication Plan

Two articles are expected to be submitted for publication by December 2024.

27. Original Data Sharing Plan

Data will be made publicly available on the Guangzhou Sport University research platform within one year after the experiment's completion.

28. Post-Trial Treatment and Management of Participants

After the trial, participants will receive corrective training for functional movements and access to training facilities.
