# Supplementary material for: The impact of 6-week flywheel eccentric training on sprint speed and change-of-direction of female basketball players
Source: PLoS One. 2025 Oct 31;20(10):e0335593. doi: 10.1371/journal.pone.0335593 (PMC12578200; doi:10.1371/journal.pone.0335593)
Supplement: S5 File — (PDF) [file pone.0335593.s007.pdf]

飞轮离心深蹲训练对大学生女子篮球运动员专项弹跳能力影响的研究方案

1. 诚信申明

本研究保证操作严格按照试验规程和数据记录的真实性；不存在任何利益冲突。

2. 研究题目

飞轮离心深蹲训练对大学生女子篮球运动员专项弹跳能力的影响

3. 经费来源

自筹经费

4. 研究事项执行流程时间安排

2024.04.15-2024.6.15 测试和干预阶段及收集数据资料

2024.06.16-2024.12.30 数据收集、统计分析，撰写论文投稿发表

5. 研究背景

纵观篮球运动的发展历程，各项技术都在不断突破、不断进步。从最初的阵地进攻到如今的快攻反击、半场攻守转换等。篮球运动在攻防速度、对抗强度等各个维度上都取得了长足进步。为了适应现代篮球比赛的节奏和要求，球员需要具备优秀的身体素质和出色的运动表现。在进攻端，球员需要快速突破对手的防线、快速传球和投篮，要求球员具备出色的运球技巧、投篮准确性和广阔的传球视野。在防守端，要求具备出色的防守技巧和意识、更好地干扰对手的进攻，同时具备快速抢断、盖帽和抢夺篮板、跟踪对手的移动的能力。因此，全面提升运动表现在训练中占有重要地位。

结合现代篮球项目特征，将运动生理学、解剖学和专项技术有机地结合起来，加强爆发力、速度和灵敏素质，以全面提高运动员的运动表现具有重要意义。在跳跃、变向等离心过程中，肌肉要承受相应的负荷压力，将其弹性势能存储起来以完成制动动作。当在动作处于向心阶段时，拮抗肌的向心收缩速度会加快，从而提高肌肉收缩速度。因此，离心训练是一种有效的训练方法，可以帮助篮球运动员提高运动表现和竞技水平。

骨骼肌的收缩可分为三种基本形式：离心收缩，向心收缩和等长收缩,三种方式向心、离心、等长收缩在骨骼肌收缩时可以缩短，达到静息长度的 50%，同时也可以拉长，达到静息长度的 170%。在日常生活和力量训练中，离心收缩皆有体现。飞轮训练是近些年兴起的一种超负荷离心抗阻训练的方法，伸手国内外学者们的欢迎。它独特的悠悠球设计，通过拉动缠绕在转轴上的带子使飞轮开始旋转，将肌肉做向心激活的动能传递给飞轮。当传动带被拉到最大长度时，飞轮继续旋转并在轴上再次缠绕传动带，向心阶段结束，肌肉即刻做离心运动，从而产生向心-离心的循环效果。

飞轮训练现有研究主要集中在对球类运动员运动表现和预防肌肉损伤方面。对于运动员，长期飞轮训练可以有效提高肌肉力量和爆发力。同时，飞轮训练还可以帮助运动员提高动作的准确性和稳定性，从而改进技术动作、减少受伤的风险。对于健康人群，长期飞轮训练能够有效改善神经肌肉能力，而对于与肢体废用和老龄化相关的疾病患者来说，飞轮训练可以恢复其肌肉功能，增强身体的平衡性和稳定性，从而减少跌倒等意外事件的发生。国外现有相关研究证明，飞轮训练对于运动表现的发展具有积极的效果。这些研究涵盖了不同类型运动员和不同运动项目，例如，飞轮训练可以增强足球运动员的冲刺和跳跃能力，提高篮球运动员的投篮和控球技巧等。相比之下，国内关于利用飞轮设备进行离心训练的研究相对较少，特别是在利用飞轮设备进行离心训练方面，需要更多的研究来探讨其具体实施方法和实际效果。

本文通过利用飞轮设备进行离心训练，分析飞轮离心深蹲训练与杠铃深蹲训练两种方式对篮球运动员运动表现影响的差异，以期对未来篮球项目运动训练方法提供一定的理论与实践经验。

6. 研究目的

飞轮离心深蹲训练作为一种新型的力量训练手段，能够进行适应性阻力与离心超负荷训练。本文的研究目的是，与篮球运动员在训练中常用的杠铃深蹲训练相比，采用飞轮离心训练对篮球运动员运动表现的影响有何差异。本研究重点探讨在提升篮球运动员运动表现时，使用飞轮训练器进行深蹲训练是否比杠铃深蹲训练效果明显。

7. 研究意义与价值

当代竞技体育的比赛成绩逐步接近人类的极限，如探索出更好的训练手段与方法，就能帮助运动员保持高水平竞技能力，甚至突破瓶颈。本文从训练实践中出发，在前人的研究基础上，使用先进的仪器和科学的测试方法，探究飞轮离心训练和杠铃深蹲训练对高校女子篮球运动员运动表现的影响，通过实验数据论证两种训练手段的差别，希冀为教练员和运动员在运动训练实践中提供有价值的参考，从而更有效的增强篮球运动员的运动表现，进而提升运动员的竞技水平。

8. 研究假设与变量

研究假设：

（1）在跳跃表现中，飞轮训练可以有效改善女子篮球运动员 CMJ、SJ、助跑双脚起跳摸高测试成绩。

（2)在速度表现中，飞轮训练可以有效地提高女子篮球运动员 20m 跑成绩。

（3）在灵敏表现中，飞轮训练可以提高女子篮球运动员 505 敏捷性测试、禁区敏捷性测试成绩。

（4）在下肢最大力量表现中，飞轮训练可以提高女子篮球运动深蹲 1RM 成绩。

9. 纳入和排除标准

纳入标准：

（1）所有参与者年龄大于 18 岁；

（2）至少 3 年以上的篮球专项经验；

（3）广体运动训练学院篮球队

（4)受试者自愿加入本研究，签署知情同意书，依从性好，配合随访干预。

排除标准

（1）依从性低；

（2）受伤失访、中途退出；

（3）未能配合

10. 设计方案，设计模式图，附 SPIRIT 模版

平行设计、前瞻、双臂随机对照试验

11. 样本量估算

分两组，共招募 20 例受试者，最后实验组（n=9）和对照组（n=10）19 个受试者纳入最终统计分析。

12. 随机和隐蔽分组的方法

基线测试后进行随机分组，运用 SPSS 的随机数把参与者随机分配到两组：FET 或 BST。受试者和所有研究员均不能事先知道或决定干预对象将分配到哪一组接受治疗，也不能从已经进入的组别推测出下一个患者将分配到哪一组。随机数字表顺序编码进行简单随机化分组，随机分配方案的隐匿则由第二研究员产生随机分配序列和第三研究员确定受试对象合格性，并且由第一作者产生和保存随机分配序列的人员。分组后，参与者被专项教练口头告知她们的小组任务，一视同仁地建议在实验期间不能进行额外的抗阻训练，但可以进行正常的篮球训练。

13. 测量指标

深蹲 1RM、深蹲跳（SJ）、反向垂直纵跳（CMJ）、反应力量指数（RSI）、离心利用率（EUR）、助跑双脚起跳摸高（RVJ）、20m、505 敏捷性测试、禁区敏捷性测试。

14. 对参试者有效性认定的定义

1. 参试者有效性的定义

签署了知情同意书、筛选合格进入研究、良好依从、按方案及流程要求完成了所有干预训练的运动员。

2.参试者退出的定义

签署了知情同意书、筛选合格进入研究后在干预前、干预中、干预后随访过程中要求退出的受试者。

3.参试者剔除的定义

签署了知情同意书、进入研究但在项目监察中或研究结束后发现不负荷纳入标准、属于排除的受试者；没有按照训练要求进行干预：依从性差；未接受干预或不遵守或承担知情协议相应的责任与义务；违背研究方案；干预过程中受伤退出的受试者。

4. 参试者失访的定义

在项目结束前经多次、多方面无法联系到的受试者；没有完成方案规定观察周期的研究对象。

5. 参试者混杂的定义

所有受试者均额外参加篮球专项训练，已被考虑；

6.参试者中止的定义

项目负责人或研究者综合多方面的因素中止受试者继续进行干预的案例；研究中发现受试者出现不良时间或不良反应

7.参试者暂停的定义

填写了知情同意书后的参试者对本项目持质疑者；填写了知情同意书后的参试者有意退出对本项目者

15. 不良事件和不良反应的定义、鉴定方法和管理制度

1. 不良事件的定义

受试者签署知情同意书并入选试验开始至后测结束之间，发生任何对身体不利事件，但不一定与治疗由因果关系，均判定为不良事件。

2. 不良反应的定义

实在实验期间任一次训练课或在观察期间产生有害而非期望的、与干预有因果关系的反应。

3. 鉴定方法

发生与干预有因果关系的有害、非期望的不良事件视为不良反应；没有因果

关系的视为不良事件。

4. 管理制度

试验期间如实记录不良事件或不良反应，包括不良事件或不良反应的发生实践、严重程度、持续时间、采取措施。

16. 参试者的征募

1. 参试者征募地点：广州体育学院数字化体能实验中心

2. 征募方法：制作研究项目参试者招募海报、微信以及代表队主教练协调等招募

3. 筛选过程：

符合纳入条件的运动员作为初步筛选对象→

身体功能动作筛查无阳性病例，得分≥14→

再按照纳入、排除标准进行严格的筛选→

最后按照是否决定资源参加本研究和签署知情同意书筛选

4. 实施筛选的研究人员：由独立于本研究的第三方人员进行

17. 参试者一般信息的收集

1. 实验收集的研究人员:徐嘉敏等硕士研究生

2. 一般信息的内容：

（1）年龄、身高、体重、BMI、受伤史、训练史、运动员等级。

（1） 身体成分各指标

（2） 基线指标和观测性能变量

18. 统计分析方法

1、运用 SPSS23.0 软件进行数理统计分析。使用独立样本 t 检验比较两组受试者的基线差异，使用 Shapiro-Wilk 检验程序检验所有变量的正态性，采用 Bonferroni 事后检验对每一个结果测量进行重复测量方差分析。并验证 Mauchly 检验的球形对称性。如不服从正态，则采用 Scheirer–Ray–Hare 检验。如果数据存在显著的交互作用，进一步使用简单效应进行分析。如果数据不存在显著的交互作用，着重分析主效应，使用配对样本 t 检验分析组内差异，用独立样本 t 检验分析组间差异。采用单向随机效应模型，用 CV[32]和 ICC[33]评估重测信度，95% CI。使用自定义电子表格执行可靠性[34]。ICC 值小于 0.5 表示可靠性差，0.5 到 0.75 之间表示可靠性中等，0.75 到 0.9 之间表示可靠性好，大于 0.90 表示可靠性极好。偏 eta 方(η_p^2)用来评价测量干预效果组间差异的效应大小，组间差异程度为小(0.01≤η_p^2≤0.06)、中等(0.06≤η_p^2 ＜0.14)或大(η_p^2≥0.14)[35]。

19. 参试者管理制度

1. 在进行干预前，所有受试者必须签署书面的知情同意书；
2. 根据纳入及排除标准入组筛选受试者；
3. 注意盲法处理和保护参试者隐私及安全

20. 标本管理制度

本研究并没有对受试者进行样本采集。

21. 药品和器材管理制度

本研究并没有进行药品干预。

22. 数据管理制度

所有数据资料均有实时备份和后台记录，并分别录入表格，由第三方统一录入和分析，而数据库由徐嘉敏管理。

23. 数据安全与监察委员会的组成和工作职责

数据由不参与后续研究的第三方管理，并由伦理委员会监察。

24. 研究团队

由研究负责人、参与研究人员、研究文章撰写、测试评估者、数据收集管理员、数据统计分析专业人员、研究数据核对人员组成。

25. 知识产权

研究的所有知识产权归于广州体育学院，根据研究者对研究的贡献大小进行署名。

26. 发表计划

预计 2024 年 12 月投稿发表论文 2 篇。

27. 原始数据共享计划

实验完成后 1 年内公开在广州体育学院科研平台

28. 试验结束后对参试者的治疗和管理

试验结束后，对受试者进行身体功能动作矫正和提供场地训练设备
